# Functional structure of local connections and differentiation of cerebral cortex areas in the neonate

**DOI:** 10.1016/j.neuroimage.2024.120780

**Published:** 2024-09

**Authors:** Jesus Pujol, Laura Blanco-Hinojo, Cecilia Persavento, Gerard Martínez-Vilavella, Carles Falcón, Mireia Gascón, Ioar Rivas, Marc Vilanova, Joan Deus, Juan Domingo Gispert, Maria Dolors Gómez-Roig, Elisa Llurba, Payam Dadvand, Jordi Sunyer

**Affiliations:** a MRI Research Unit, Department of Radiology, Hospital del Mar, Passeig Marítim 25-29, Barcelona 08003, Spain; b ISGlobal, Barcelona, Spain; c Universitat Pompeu Fabra (UPF), Barcelona, Spain; d CIBER Epidemiología y Salud Pública (CIBERESP), Instituto de Salud Carlos III, Madrid, Spain; e Barcelonaβeta Brain Research Center (BBRC), Pasqual Maragall Foundation, Barcelona, Spain; f CIBER Bioengineering, Biomaterials and Nanomedicine (CIBER-BBN), Instituto de Salud Carlos III, Madrid, Spain; g IMIM (Hospital del Mar Medical Research Institute), Barcelona, Spain; h Department of Clinical and Health Psychology, Autonomous University of Barcelona, Barcelona, Spain; i BCNatal, Fetal Medicine Research Center, Hospital Sant Joan de Déu and Hospital Clínic, University of Barcelona, Barcelona, Spain; j Institut de Recerca Sant Joan de Déu, Barcelona, Spain; k Primary Care Interventions to Prevent Maternal and Child Chronic Diseases of Perinatal and Developmental Origin Network (RICORS), RD21/0012/1&3, Instituto de Salud Carlos III, Madrid, Spain; l Department of Obstetrics and Gynaecology. Institut d'Investigació Biomèdica Sant Pau - IIB Sant Pau. Hospital de la Santa Creu i Sant Pau, Barcelona, Spain

**Keywords:** Functional connectivity, Development, Neonates, cortical areas, MRI

## Abstract

Neuroimaging research on functional connectivity can provide valuable information on the developmental differentiation of the infant cerebral cortex into its functional areas. We examined healthy neonates to comprehensively map brain functional connectivity using a combination of local measures that uniquely capture the rich spatial structure of cerebral cortex functional connections. Optimal functional MRI scans were obtained in 61 neonates. Local functional connectivity maps were based on Iso-Distance Average Correlation (IDAC) measures. Single distance maps and maps combining three distinct IDAC measures were used to assess different levels of cortical area functional differentiation. A set of brain areas showed higher connectivity than the rest of the brain parenchyma in each local distance map. These areas were consistent with those supporting basic aspects of the neonatal repertoire of adaptive behaviors and included the sensorimotor, auditory and visual cortices, the frontal operculum/anterior insula (relevant for sucking, swallowing and the sense of taste), paracentral lobule (processing anal and urethral sphincter activity), default mode network (relevant for self-awareness), and limbic-emotional structures such as the anterior cingulate cortex, amygdala and hippocampus. However, the results also indicate that brain areas presumed to be actively developing may not necessarily be mature. In fact, combined distance, second-level maps confirmed that the functional differentiation of the cerebral cortex into functional areas in neonates is far from complete. Our results provide a more comprehensive understanding of the developing brain systems, while also highlighting the substantial developmental journey that the neonatal brain must undergo to reach adulthood.

## Highlights

• The study informs on the differentiation of cerebral cortex areas in neonates.
• A novel approach was used to map the functional structure of local connections.
• The existence of measurable connections does not necessarily indicate full maturity.
• A long developmental journey the neonatal brain must undergo to reach adulthood.

## 1 Introduction

During the early days of life, individuals embark on a challenging interaction with the external environment. It is crucial for the neonate to possess the ability to coordinate basic motor activities, engage in active feeding, express basic needs affectively, learn from experience, maintain adequate sensory contact, and establish a primary sense of self (Lagercrantz and Changeux, 2010; von Hofsten and Rosander, 2018). The brain systems supporting such a repertoire of adaptive behaviors in neonates need to be sufficiently mature to successfully navigate this critical stage of development.

Neuroimaging research on functional connectivity has provided valuable information as to the developing cerebral cortex areas and level of organization of the neonatal brain. In general, it is implicitly assumed that when connectivity is patent, the implicated brain structure is active and to some extent competent in executing dedicated neural operations (Grayson and Fair, 2017; Gao et al., 2017; Karolis et al., 2023). Relevant research has contributed to identifying the developing brain connections in neonates (e.g., Doria et al., 2010; Smyser and Neil, 2015; Gao et al., 2017; Zhang et al., 2019; Peng et al., 2020; Eyre et al., 2021; Liu et al., 2023; Karolis et al., 2023). Network configuration begins early in the fetal stage, prior to ex-utero exposure. Interhemispheric coupling is measurable by the third trimester, as are the connections between each major hemisphere division (dorsal-ventral, medial-lateral and anterior-posterior) (Karolis et al., 2023). In neonates, most basic resting-sate networks are topographically identifiable. Network assembly is more complete in the primary sensorimotor, visual and auditory cortices than in association areas and default mode network (Gao et al., 2017; Eyre et al., 2021). Network remodeling remains notably active during the first two years of life, with different maturation timelines for distinct hierarchical functional systems (Liu et al., 2023).

Despite the contributions from functional connectivity studies, the functional anatomy of the brain systems supporting neonatal behavior has not been fully characterized. Moreover, the maturation process of a neural structure is not complete but may only be just beginning when functional connectivity can first be demonstrated. Brain areas active during the neonatal stage obviously continue to mature until late development (Dosenbach et al., 2010; Grayson and Fair, 2017; Pujol et al., 2021; Liu et al., 2023; Wang et al., 2023).

Large-scale, long-distance functional connectivity measures have been used to assess the overall differentiation of the brain into its various systems (Grayson and Fair, 2017; Gao et al., 2017; Eyre et al., 2021; Nielsen et al., 2023). Similarly, the combination of short-range measures can inform the connectivity-related specialization of cerebral cortex areas (Macià et al., 2018; Pujol et al., 2019a; 2021; 2023). We expanded well-established MRI measures of local functional connectivity (Zang et al., 2004; Sepulcre et al., 2010; Tomasi and Volkow, 2010) by combining “Iso-Distance Average Correlation” (IDAC) measures across three local distances. The IDAC measures represent the average functional MRI temporal correlation of a given brain unit, or voxel, with other units located at increasingly separated iso-distant intervals (Macià et al., 2018).

Our approach differs from other functional MRI studies of local synchronicity by using multi-distance local measures rather than relying on a single local measure. The combined measures can uniquely capture the rich spatial structure of cerebral cortex functional connections and offer a detailed mapping that has successfully discriminated between major classical anatomo-functional cortical areas in adults (Macià et al., 2018; Pujol et al., 2019a; 2021; 2023). While conventional functional connectivity measures have identified brain systems presumed to be actively developing, our multi-distance local functional connectivity mapping can provide insights as to the differentiation of the cerebral cortex into its functional areas. This type of information is currently lacking in neonates.

Previous findings suggest that the differentiation of short connections in cortical areas continues until adulthood (Dosenbach et al., 2010; Ouyang 2017; Pujol et al., 2021). We anticipate that the use of multi-distance local connectivity mapping would help to differentiate between simply actively developing and fully mature cortical areas, and thus better capturing the immature nature of the cerebral cortex in neonates.

In this study, we examined a group of healthy infants at the end of the neonatal stage to map the functional structure of cerebral cortex local connections using IDAC measures. The aim of this research was to provide a more comprehensive understanding of the actively developing brain systems in neonates and to originally map the emergent differentiation of cerebral cortex into its functional areas.

## 2 Methods

### 2.1 Study population

This study was conducted as part of the European Research Council-funded project AirNB: Prenatal exposure to urban air pollution and pre- and post-natal brain development (ERC- Advanced Grants 2018, agreement 785,994). The primary objective of the AirNB project is to assess the impact of prenatal environmental factors on brain development. The project involves a cohort of 1080 mother-child pairs recruited between 2018 and 2021. A subset of this cohort participated in the MRI substudy.

For the MRI sample, recruitment was consecutive and based on the parents' agreement to participate. During the prenatal 32-week gestation visit, all eligible cases from the general cohort were presented with a comprehensive description of the study's aims and procedures. Exclusions for the MRI substudy included cases of premature birth (≤37 weeks of gestation), any medical disorder or respiratory risk, postnatal surgery, congenital malformation, and formal contraindication to MRI. Consent for examination was obtained for 132 neonates and resting-state functional MRI of optimal quality was obtained in 61 cases. The sample consisted of 32 females and 29 males, with a mean postnatal age of 29.5 days and a standard deviation (SD) of 4.1 days. The corresponding mean postmenstrual age was 44.1 weeks (SD: 1.2 weeks, range: 41.0 to 46.1 weeks). Demographic details are provided in Table 1.

**Table 1:** Demographic details of the study sample.

Sex, F	32 (52.5 %)
Gestational age at birth, wks	39.9 ± 1.0 (37.4 – 41.8)
Weight at birth, g	3297 ± 368 (2480 – 4170)
Head circumference at birth, cm	34.8 ± 2.2 (32.0 – 49.0]
Postnatal age at MRI scan, d	29.5 ± 4.1 (23 – 42)
Gestational age at MRI scan, wks	44.1 ± 1.2 (41.0 – 46.1)
Maternal age at 1st trim., yrs	34.4 ± 3.8 (27 – 44)
Maternal education	
Primary education	0 (0 %)
Secondary studies	14 (23 %)
University, Postgraduate studies	47 (77 %)
Family income, €/yr	47,932 ± 10,457 (27,949 – 82,824)

Data are given as No (%), or mean ± SD (range).

The study received approval from the Ethical Committee of the Parc de Salut Mar, Barcelona, (CEIm 2018/8050/I.). The study adhered to the guidelines set forth in the Declaration of Helsinki. Informed consent was obtained from a parent and/or legal guardian for each case. No compensation was provided to the parents of the assessed neonates. Data will be available via a request to the Authors with no particular restrictions, although a formal data sharing agreement will be considered.

### 2.2 MRI acquisition

MRI scans were acquired while the neonate was asleep, without the administration of any sedation. The early weeks of life provide a valuable opportunity for this, as infants easily fall asleep after being fed and often remain asleep throughout the MRI acquisition. An MRI-adapted foam cradle, double hearing protectors, TV monitor and a pediatric pulse oximeter were used to ensure a secure acquisition.

The MRI protocol consisted of several structural sequences, including high-resolution 3D anatomical images, diffusion tensor imaging (DTI), and fluid-attenuated inversion recovery (FLAIR) imaging, as well as the resting-state functional MRI, which serves as the basis for the current analysis. Among the 132 neonates for whom permission was obtained, natural sleep was achieved and allowed us to acquire at least one MRI sequence in 109 cases. However, successful acquisition of the noisy functional MRI sequence was less frequent (i.e., 48 neonates woke up before completing the functional sequence), resulting in optimal-quality images suitable for analysis in 61 cases.

Functional MRI was collected on a 3.0 Tesla scanner (Ingenia CX, Philips Healthcare, Best, The Netherlands) equipped with a 32-channel head-coil using a conventional single-shot, gradient-echo echoplanar imaging (EPI) sequence. Acquisition parameters were set as repetition time, 2000 msec; echo time, 35 msec; pulse angle, 70°; 18-cm field of view; 64 × 64-pixel matrix; slice thickness 2.8 mm (slice gap 0 mm); acquisition voxel size 2.8 × 2.8 × 2.8 mm. Forty-six slices were acquired to generate 177 whole-brain volumes, excluding initial additional 3 dummy scans (total acquisition duration of 6 min).

### 2.3 MRI analysis

Obtaining optimal group-level functional connectivity maps of the neonatal brain poses challenges due to factors such as the small size, thinner cortex, reversed contrast compared to adults and the underdeveloped morphology of certain gyri and sulci. Inaccurate brain tissue segmentation and spatial transformation to a model can indeed distort the functional anatomy and underestimate the details of the original individual data (Gilmore et al., 2018; Fitzgibbon et al., 2020). In this study, functional connectivity measures were computed in native space with minimal image preprocessing and without gray/white matter tissue segmentation. Also, an individual analysis was conducted to validate the findings identified in the group maps.

#### 2.3.1 Preprocessing

The imaging data were processed using Statistical Parametric Mapping software (SPM12, The Wellcome Department of Imaging Neuroscience, London) running on MATLAB version 2017a (The MathWorks Inc. Natick, Mass) and tools from the FMRIB's Software Library (FSL; version 6.0.5.1, http://www.fmrib.ox.ac.uk/fsl). The functional images were realigned to the mean time series, resliced to a resolution of 2 × 2 × 2 mm and smoothed by convolving the image with a 4 × 4 × 4 mm full width at half maximum (FWHM) Gaussian kernel. Motion-affected image volumes were discarded using conventional scrubbing procedures (Power et al., 2014) detailed in the Supplementary Material.

#### 2.3.2 Iso-Distant average correlation (IDAC) maps

The steps employed to generate the cerebral cortex IDAC maps in adults have been previously reported (Macià et al., 2018). Below is a description of the procedure adapted for the neonatal brain.

IDAC measures were estimated in native space. Computations were conducted within a whole-brain mask that was split into left and right hemispheres. This step ensured that adjacent voxels from the medial regions of one hemisphere were not locally associated with those from the other hemisphere. IDAC maps were generated by calculating the average temporal correlation of each voxel with its neighboring voxels placed at increasingly separated Euclidean iso-distant intervals (the definition and mathematical formulation are provided in the Supplementary Material). The analyses were adjusted by including 6 rigid body realignment parameters, their first-order derivatives and the global brain signal as regressors. All functional MRI time series were band-passed with a Discrete Cosine Transform (DCT) filter, allowing frequencies in the 0.01–0.1 Hz interval to pass through. Three IDAC maps were obtained at distance intervals of 1–4 mm, 4–7 mm, and 7–10 mm. These distance intervals mirror those utilized in previous adult studies (5–10 mm, 15–20 mm, and 25–30 mm) (Macià et al., 2018; Pujol et al., 2019a; 2023), adjusted for the smaller size of the neonatal brain. Indeed, the brain volume of neonates is roughly one-third the volume of the adult brain (Gilmore et al., 2018). The two hemispheres were merged back together once the IDAC values had been calculated.

#### 2.3.3 Spatial transformation to a common space

A study-based template was created from the original functional MR images of all participants using specific tools detailed in the Supplementary Material. The IDAC maps, estimated in native space, were registered to this study's functional MRI template. Subsequently, the transformed IDAC maps were averaged to generate a mean IDAC image for each distance. Finally, the native IDAC maps were registered to these mean IDAC images.

#### 2.3.4 Individual analysis

This analysis involved identifying cortical areas with relatively high local functional connectivity values for each neonate and distance map. These areas were operatively defined as those exhibiting functional connectivity values one standard deviation above the mean of the brain (i.e., Cohen effect size of 1 (Cohen, 1992).

#### 2.3.5 Group analysis

Once transformed to the common space, the individual IDAC maps were included in a random-effects group ANOVA analysis using the SPM full-factorial model and distance-specific grand mean scaling. One-sample *t*-test maps were generated for each connectivity distance, and paired *t*-test maps were used to confirm significant effects across distances within the regions of interest. Results were considered significant when voxels survived whole-brain family-wise error (FWE) correction (*p* < 0.05), as calculated using SPM.

Multi-distance IDAC color maps were obtained by overlaying the three one-sample IDAC maps using RGB color coding. The RGB color channels enabled the simultaneous display of three values, with RED representing the results from the 1–4 mm IDAC map analyses, GREEN representing 4–7 mm, and BLUE 7–10 mm. The overlapping of these primary colors can generate a full range of secondary colors.

## 3 Results

**Fig. 1:**
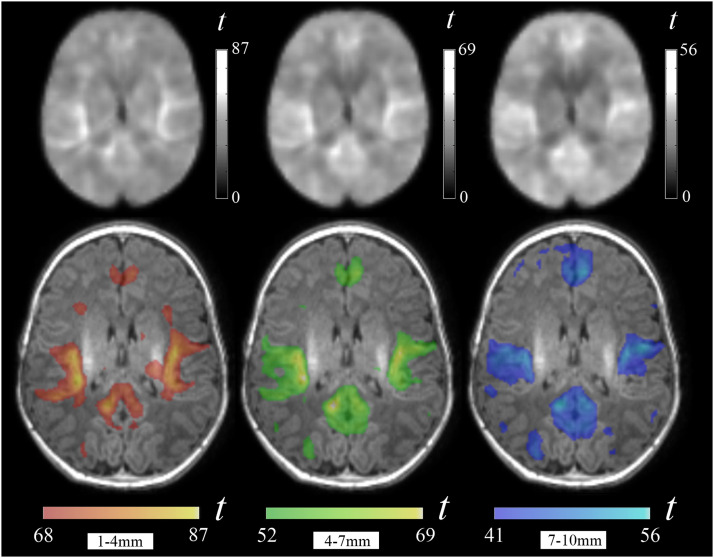
One-sample t maps from the group analysis of local functional connectivity for each distance. The bottom images highlight cortical areas exhibiting the highest local connectivity, surpassing the brain mean by one standard deviation. The right hemisphere is shown in the right side of the images.

The group analysis of the three local functional connectivity distances generated three one-sample *t*-test maps characterized by showing discrete regions with connectivity higher than the rest of brain parenchyma. Thresholding the *t*-test maps at brain mean plus one standard deviation served to emphasize the areas with more developed local functional connectivity (Fig. 1).

**Fig. 2:**
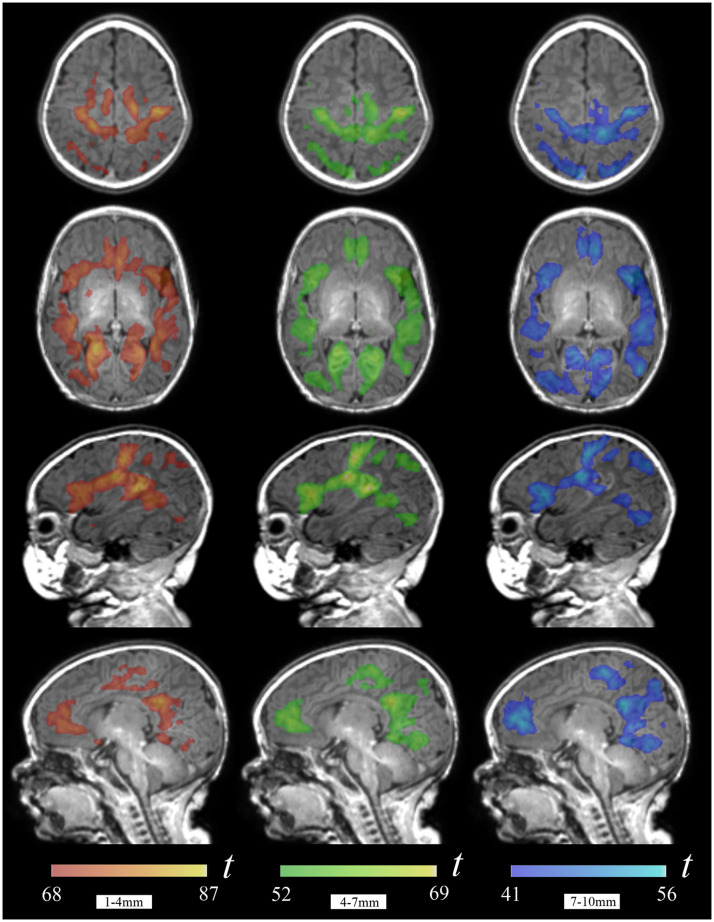
Group one-sample t maps for short (1–4 mm), middle (4–7 mm) and long (7–10 mm) local functional connectivity. The figure illustrates brain areas with higher connectivity values (above the brain mean plus one standard deviation). It is remarkable that there are notable anatomical similarities across the three distance maps. The right hemisphere is shown in the right side of axial images. The opercular region view (third row) corresponds to the right hemisphere and the brain medial view (fourth row) to the left hemisphere.

The group maps in Fig. 2 show the cortical areas that were identified practically in each neonate in the individual analysis (Supplementary Table 1, Supplementary Fig. 1). This set of areas included the sensorimotor cortex strip with a cranial extension to the paracentral lobule, the visual cortex in the occipital lobe and middle temporal (MT) visual area, the auditory cortex in the superior temporal gyrus extending to the posterior insula, the frontal operculum/anterior insula, the posterior cingulate cortex/precuneus, and anterior medial frontal cortex.

The group maps in Supplementary Fig. 2 show additional brain structures that were identified in >50 % of cases in the individual analysis (Supplementary Table 1). These structures included the parietal association cortex, right anterior cingulate cortex, olfactory cortex, amygdala and anterior hippocampus.

The results were generally symmetrical, with similar findings for the right and left hemispheres except for the anterior cingulate cortex, which showed more frequently developed connectivity in the right hemisphere (Supplementary Fig. 3). For instance, in the short-distance (1–4 mm) analysis, IDAC values one standard deviation above mean were observed in 72 % of cases in the right hemisphere compared with 21 % in the left hemisphere (χ^2^= 31.6; *p* < 0.0001) (Supplementary Table 1).

**Fig. 3:**
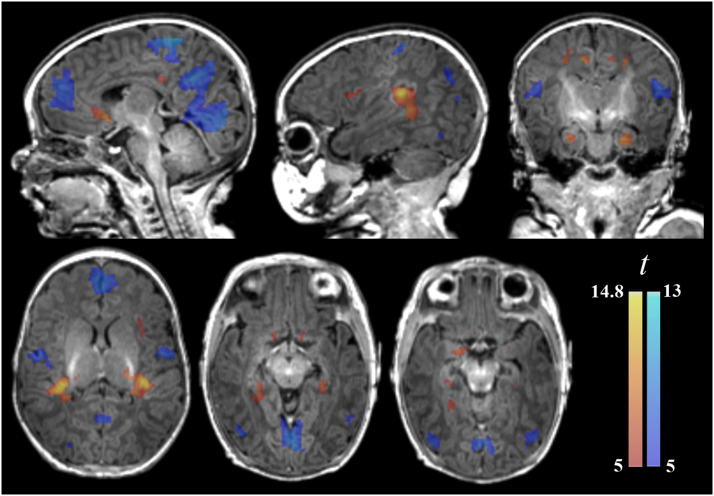
Brain areas with a significant distance effect in paired *t*-tests. The hot color display represents areas with higher short-distance (1–4 mm) functional connectivity compared to long-distance (7–10 mm), while the cold color display represents areas with higher long-distance functional connectivity compared to short-distance. The right hemisphere is shown in sagittal and coronal images and in the right side of axial images.

A relevant characteristic of local connectivity in the immature brains of neonates was the remarkable anatomical resemblance of the three distance maps (Figs. 1, 2). However, some significant distance effects were demonstrated. Areas showing stronger short-distance functional connectivity than long-distance connectivity were identified in the posterior extent of the auditory gyrus of Heschl, the insula, posterior and anterior cingulate cortex, olfactory cortex, amygdala, and anterior hippocampus. Conversely, stronger long-distance connectivity than short-distance connectivity was observed in the visual cortex, precuneus, anterior medial frontal cortex, paracentral lobule, sensorimotor cortex and opercular region (Fig. 3, Supplementary Table 2). No differences in the results were observed after adjusting the analyses by age, sex or both (Supplementary Table 3).

**Fig. 4:**
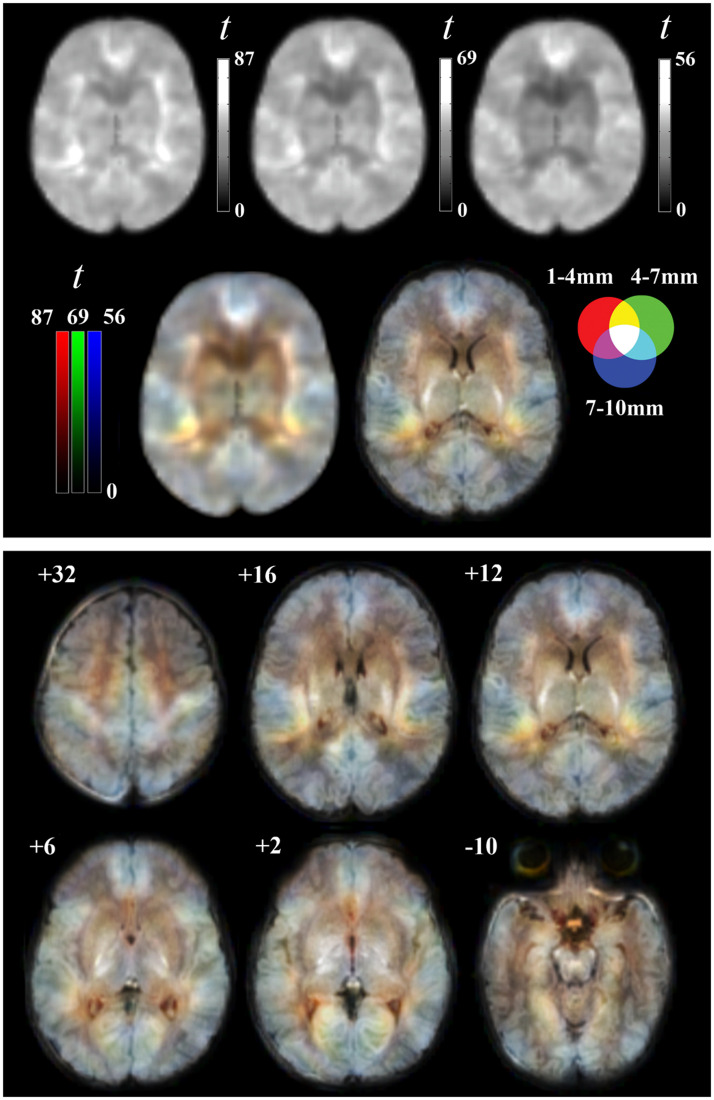
Composite (RGB) Iso-Distant Average Correlation (IDAC) brain mapping in neonates. Top panel: the gray images show one-sample t maps for the three distances, with each map scaled to the conventional 0–255 gray range. The color images show the result obtained by superimposing the three IDAC maps using RGB (red, green, and blue) display, both without and with the overlay of the corresponding anatomic image. Bottom panel: six representative axial slices at different distances (in mm) from the anterior commissure. The right hemisphere is shown in the right side of the images.

**Fig. 5:**
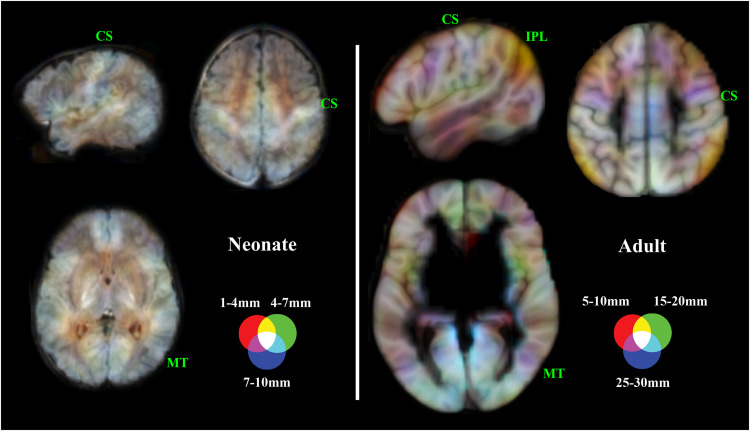
Composite RGB brain mapping of local functional connectivity in the group of neonates and equivalent adult maps from a previously reported sample (Pujol et al., 2021). In neonates, structures with similar local connectivity across distances (tending to white on the map) stand out, while the differentiation of cortical areas within these structures (depicted in color) is limited. In contrast, local connectivity in adults exhibits a large degree of distance specificity and effectively discriminates between major cortical area (maps generated from a group of 121 healthy adults with a mean age of 34.6 years, SD of 10.2 years, range 16 to 61 years, male/female: 66/55). CS refers to the central sulcus, MT to the middle temporal visual area and IPL to the inferior parietal lobule. The right hemisphere is shown in sagittal images and in the right side of axial images.

The combined RGB display of three local distance maps served to illustrate the second-order, multi-distance differentiation of cerebral cortex into functional areas (Fig. 4). In the composite maps of neonates, a set of areas showed uniformly high connectivity values across all three RGB channels, along with the significant but incipient effect of both short and long distances. In contrast, in the mature adult brain, local connectivity measures effectively distinguished the major classical anatomo-functional cortical areas, as it was shown in an early study (Pujol et al., 2021) that used an equivalent imaging approach (Fig. 5).

## 4 Discussion

Our study involved examining a group of healthy infants at the end of the neonatal stage of development to map the functional structure of local connections within the cerebral cortex. The generated maps for each short-range distance successfully differentiated between brain areas with high and low functional connectivity. The detail of the maps may provide a more comprehensive understanding of the actively developing brain systems in neonates. However, the results also indicate that brain areas presumed to be actively developing may not necessarily be mature. In fact, the multi-distance RGB display of local connectivity confirms that while the functional differentiation of cerebral cortex areas in neonates is indeed initiated, it remains predominantly immature at this stage.

Our study revealed relatively high local functional connectivity in the sensory cortices, which aligns with previous findings in neonatal functional connectivity studies, especially in the somatosensory, visual, and auditory domains (Doria et al., 2010; Smyser and Neil, 2015; Grayson and Fair, 2017; Gao et al., 2017; Cao et al., 2017; Zhang et al., 2019; Peng et al., 2020; Eyre et al., 2021). In contrast, the connectivity of olfactory and gustatory areas has rarely received attention in previous research (Adam-Darque et al., 2021). Nonetheless, our study observed a relatively high local functional connectivity in each neonate in the frontal operculum/anterior insula region, which houses the cortical representation of the gustatory sense (de Araujo and Simon, 2009; Dagher, 2012). Our data, therefore, are consistent with the early emergence of the sense of taste (Ventura and Worobey, 2013). However, it is important to note that the role of this brain region extends beyond the sense of taste, as it supports other vital functions in the neonates, such as sucking, swallowing and crying (Barlow, 2009; Mistry and Hamdy, 2008). Consequently, it is unsurprising that the results from the oral cortex prominently feature in our maps, given its outstanding role in the behavior of neonates.

Another noteworthy finding is related to the paracentral lobule, a brain region located on the medial surface of the cerebral hemisphere containing the cortical representation of the lower limbs and anogenital area in the sensory, motor and supplementary motor homunculus (Parpia, 2011; Cazala et al., 2015). At this developmental stage, its relevance may be related in part to the anal and urethral sphincters. Although voluntary sphincter control is immature, sphincter sensations may arguably play an important role in the newborn's body awareness (Nevéus and Sillén, 2013; Jansson et al., 2005). Thus, it is conceivable that the paracentral lobule is functionally organized for basic sensory operations soon after birth.

The default mode network is composed of interconnected brain areas that are highly organized in adults and play a central role in self-awareness (Davey et al., 2016). Previous studies utilizing a variety of functional connectivity metrics have indicated the presence of a primitive version of the default mode network in newborns (Gao et al., 2009; 2015; Grayson and Fair, 2017). In our study, we identified two major elements of the network – the anterior medial frontal cortex and the posterior cingulate cortex – with a high degree of short-range functional connectivity in almost every neonate. However, the angular gyrus, another key element, was less prominent in our maps. These findings, combined with evidence from larger-scale network studies (Grayson and Fair, 2017; Gao et al., 2017), suggest that the default mode network undergoes active construction during the neonatal period. It has been proposed that infants exposed to early adversity are vulnerable to disruptions in information flow within the network, particularly between the medial frontal cortex and the posterior cingulate (Pujol et al., 2019b). Such a breakdown in the default mode network could potentially contribute to the development of psychopathic personality traits (Pujol et al., 2019b; 2012).

Furthermore, we identified several elements of the limbic and paralimbic domains in a relevant number of neonates, including the amygdala, anterior hippocampus, olfactory cortex, anterior cingulate cortex and anterior insula. These structures were not collectively identified in previous neonatal studies, with few exceptions (e.g., Graham et al., 2016; Liu et al., 2021; Scheinost et al., 2022). However, all are elements potentially supporting distinct aspects of the neonatal instinctive and affective behaviors. Notably, we observed an asymmetry in the anterior cingulate cortex, indicating higher local functional connectivity in the right hemisphere. An early study linked the anatomy of the right anterior cingulate cortex, but not the left, to "harm avoidance," a basic temperament trait (Pujol et al., 2002). The current functional connectivity findings provide new imaging evidence supporting the classical notion that core temperament traits are established at a very early age (Rothbart and Bates, 2006).

Beyond contributing to our understanding of the developing brain systems, our imaging approach allowed us to illustrate a second-level differentiation of cortical areas. Overall, the maps underscore the immaturity of the neonatal brain. While there were significant differences in relative connectivity strength between short and long local distances, the composite maps were largely unable to distinguish most classical anatomical and functional cortical areas. This contrasts sharply with the functional area parcellation achieved in adult brains through the combination of three short-range connectivity measures. Previous studies demonstrated the ability of adult maps to differentiate, for example, primary sensory areas, middle temporal visual area (MT), premotor cortex, prefrontal cortex, elements of the default mode network, and the inferior parietal lobule (Macià et al., 2018; Pujol et al., 2021; 2023).

IDAC measures implicitly provide information on the functional status of local cortical circuits, and their combination uniquely captures their rich functional structure. It is relevant to consider that the local measures inform on the activity of both pyramidal (principal) neurons and inhibitory interneurons. We have previously observed that the IDAC measures are sensitive to the gamma-aminobutyric acid (GABA) agonist benzodiazepine alprazolam (Blanco-Hinojo et al., 2021). In general, the excitation/inhibition balance expressed in functional connectivity measures is modulated by the GABA agents (Zhang et al., 2024). Moreover, the distribution of IDAC alterations in patients with schizophrenia is consistent with the cortical distribution of parvalbumin and somatostatin GABA interneurons, further supporting the evidence of a deficient GABA system in this disorder. The neonatal period is critical for the maturation of the interneuron system to establish an adequate excitation/inhibition balance (Kirmse and Zhang, 2022). Our cross-sectional data can provide insight into the functional status of local circuits at the end of the neonatal period. Nevertheless, it would be highly relevant to conduct longitudinal follow-ups to track how these connectivity patterns change over time or predict developmental outcomes.

One of the main limitations of our study pertains to the challenge of obtaining anatomically accurate group results for functional connectivity measures in the immature brains of neonates (Gilmore et al., 2018; Fitzgibbon et al., 2020). To address these technical limitations, we estimated local functional connectivity measures in native space after minimal image preprocessing, and individual analyses were conducted to validate the findings identified in the group maps. Additionally, we chose not to quantitatively compare neonatal and adult brains due to their non-equivalent anatomy in terms of gray/white matter contrast, cortical thickness, and the degree of morphological development in association areas. Hence, we considered a qualitative appraisal to be more appropriate for illustrating the developmental gap between neonatal and adult brains.

In conclusion, our examination focused on two levels of functional differentiation in the neonatal cortex. Firstly, certain cortical areas exhibited a higher degree of local functional connectivity, regardless of distance, differentiating them from the rest. Similar to previous studies, our results identified brain systems with presumed active development during the neonatal stage. The detailed maps may contribute to the identification of the neural basis for the basic repertoire of neonatal behavior. In addition, our multi-distance local functional connectivity mapping provides insight as to the differentiation of the cerebral cortex into functional areas. However, these maps strongly emphasize that the existence of measurable connections do not necessarily indicate full maturity of cerebral cortex areas, highlighting the substantial developmental journey that the neonatal brain must undergo to reach adulthood.

## Financial support

The AirNB project has received funding from the European Research Council (ERC) under the European Union's Horizon 2020 research and innovation programme (AirNB projectERC- Advanced Grants 2018, agreement 785994).

## CRediT authorship contribution statement

**Jesus Pujol:** Writing – review & editing, Writing – original draft, Validation, Supervision, Investigation, Data curation, Conceptualization. **Laura Blanco-Hinojo:** Writing – review & editing, Methodology, Formal analysis. **Cecilia Persavento:** Writing – review & editing, Investigation, Data curation. **Gerard Martínez-Vilavella:** Writing – review & editing, Methodology, Formal analysis. **Carles Falcón:** Writing – review & editing, Investigation, Conceptualization. **Mireia Gascón:** Writing – review & editing, Investigation. **Ioar Rivas:** Writing – review & editing, Investigation. **Marc Vilanova:** Writing – review & editing, Investigation. **Joan Deus:** Writing – review & editing, Supervision. **Juan Domingo Gispert:** Writing – review & editing, Investigation, Conceptualization. **Maria Dolors Gómez-Roig:** Writing – review & editing, Supervision, Conceptualization. **Elisa Llurba:** Writing – review & editing, Supervision, Conceptualization. **Payam Dadvand:** Writing – review & editing, Supervision, Conceptualization. **Jordi Sunyer:** Writing – review & editing, Supervision, Resources, Methodology, Funding acquisition, Conceptualization.

## Declaration of competing interest

The authors declare no conflict of interest.

## Data Availability

Data will be made available on request.

## Appendix Supplementary materials

Image, application 1
