# Supplementary material for: Functional structure of local connections and differentiation of cerebral cortex areas in the neonate
Source: Neuroimage. 2024 Sep;298:120780. doi: 10.1016/j.neuroimage.2024.120780 (PMC11399311; doi:10.1016/j.neuroimage.2024.120780)
Supplement: Supplementary file 1 [file mmc1.pdf]

Functional structure of local connections and differentiation of cerebral cortex areas in the neonate

Supplementary Material

Supplementary Methods

Supplementary Figures

Supplementary Tables

Supplementary Methods

Functional MRI quality control

The parameters resulting from functional MRI realignment were used for scrubbing, namely, discarding motion-affected volumes (Power et al., 2014). For each subject, mean inter-frame motion measurements (Pujol et al., 2014) served as an index of data quality to flag volumes of suspect quality across the run. Volumes with mean inter-frame motion > 0.3 mm, the immediately preceding and the succeeding two volumes were all discarded. Using this procedure, a mean of 5.1 (SD, 9.3; range 0-47) volumes from the total of 177 volumes included in the functional MRI sequence were removed in the final sample.

Definition of Iso-Distant Average Correlation (IDAC)

A novel mapping was used to characterize the functional structure of the cerebral cortex based on Iso-Distant Average Correlation (IDAC) measures. Essentially, IDAC mapping expands well-established MRI measures of local functional connectivity (Sepulcre et al., 2010; Tomasi and Volkow, 2010; Zang et al., 2004) by combining the connectivity maps of varying distances. Composite IDAC maps may uniquely inform the connectivity-related specialization of the cerebral cortex as local connectivity is distance-specific to a large extent and proved to discriminate well between major classical anatomo-functional cortical areas (Macià et al., 2018; Pujol et al., 2019).

We defined the concept of "Iso-Distant Average Correlation" (IDAC) to describe the pattern of correlation decay in the close vicinity of a voxel (Macià et al., 2018). $IDAC_i(h)$ was consequently defined as the average temporal correlation of voxel *i* with all the voxels located at a given Euclidean distance interval *h*. Functional MRI data sets being a discrete sample, any distance interval h must be necessarily transformed into a discrete iso-distant interval $H_k=(h_k, h_{k+1})$, with h_k being a set of successively increasing distances covering the whole vicinity of a given voxel.
The set of iso-distant intervals H_k were selected so that temporal correlations were mainly positive, decreased monotonically and in which horizontal axon collaterals were considered likely to form local networks. For the present study, we defined 3 iso-distant intervals: 1-4mm, 4-7mm and 7-10mm, with constant thicknesses but increasing number of voxels.
We first computed a correlation matrix *C* of Pearson coefficients comparing the functional MRI signal time course of all the voxels in our study mask with each other's.

$$C_{i,j} = \frac{\sum_{k=1}^{M}\left(Y_{i,k}-\overline{Y_i}\right)\cdot\left(Y_{j,k}-\overline{Y_j}\right)}{\sqrt{\sum_{k=1}^{M}\left(Y_{i,k}-\overline{Y_i}\right)^2}\cdot\sqrt{\sum_{k=1}^{M}\left(Y_{j,k}-\overline{Y_j}\right)^2}}$$

where M is the length of the functional MRI signal time series and i and j index all the voxels entering our study mask. We then transformed the Pearson correlation matrix C into a Gaussian distributed z-score correlation matrix Z by applying a Fisher transform.

$$Z_{i,j} = \frac{\sqrt{M-3}}{2} \cdot \ln\left(\frac{1+C_{i,j}}{1-C_{i,j}}\right)$$

We obtained then $IDAC_i(h_k)$ by averaging the correlation coefficients of voxel i with all the voxels j belonging to the interval H_k.

$$IDAC_i(h_k) = \frac{\sum_{j \epsilon H_{k,i}} Z_{i,j}}{N_{k,i}}$$

In short, IDAC values are defined as the mean correlation z-score between one voxel's functional MRI signal and the functional MRI signal of all the voxels within the iso-distant interval $H_{k,i}$. Note that, for a given distance interval k, the number of voxels within the concentric iso-distant interval $N_{k,i}$ is not necessarily the same for every voxel i due to the edge effects of the study mask.

Spatial transformation

A study-based template was generated from the functional MR images of all participants using the antsMultivariateTemplateConstruction2.sh script in the Advanced Normalization Tools (ANTs, http://stnava.github.io/ANTs/) software package with a cross correlation similarity metric and a Greedy SyN transformation model used for non-linear registration (Avants et al., 2008). The parameters used to align each scan also included two template construction iterations, 0.2 gradient step size, maximum 1000x500x250x100 multi-resolution iterations per registration, shrink factors 8x4x2x1, and smoothing factors 3x2x1x0.

IDAC maps generated in native space were diffeomorphically and affine transformed to the study template firstly and then to group mean IDAC images using the diffeomorphic symmetric image normalization method (SyN) in ANTs with the antsRegistrationSyN and antsApplyTransform commands with default settings.

Supplementary References

Power JD, Mitra A, Laumann TO, Snyder AZ, Schlaggar BL, Petersen SE. Methods to detect, characterize, and remove motion artifact in resting state fMRI. Neuroimage. 2014 Jan 1;84:320-41.

Pujol J, Macià D, Blanco-Hinojo L, Martínez-Vilavella G, Sunyer J, de la Torre R, Caixàs A, Martín-Santos R, Deus J, Harrison BJ. Does motion-related brain functional connectivity reflect both artifacts and genuine neural activity? Neuroimage. 2014 Nov 1;101:87-95.

Sepulcre J, Liu H, Talukdar T, Martincorena I, Yeo BT, Buckner RL. The organization of local and distant functional connectivity in the human brain. PLoS Comput Biol. 2010;6(6):e1000808.

Tomasi D, Volkow ND. Functional connectivity density mapping. Proc Natl Acad Sci U S A. 2010;107(21):9885-9890.

Zang Y, Jiang T, Lu Y, He Y, Tian L. Regional homogeneity approach to fMRI data analysis. Neuroimage. 2004;22(1):394-400.

Macià D, Pujol J, Blanco-Hinojo L, Martínez-Vilavella G, Martín-Santos R, Deus J. Characterization of the spatial structure of local functional connectivity using multi-distance average correlation measures. Brain Connectivity 2018 Jun;8(5):276-287.

Pujol J, Blanco-Hinojo L, Maciá D, Alonso P, Harrison BJ, Martínez-Vilavella G, Deus J, Menchón JM, Cardoner N, Soriano-Mas C. Mapping alterations of the functional structure of the cerebral cortex in obsessive-compulsive disorder. Cereb Cortex. 2019 Dec 17;29(11):4753-4762.

Avants B.B., Epstein C.L., Grossman M., Gee J.C. (2008). Symmetric diffeomorphic image registration with cross-correlation: Evaluating automated labeling of elderly and neurodegenerative brain. Med Image Anal. 12, 26-41.

Supplementary Figures

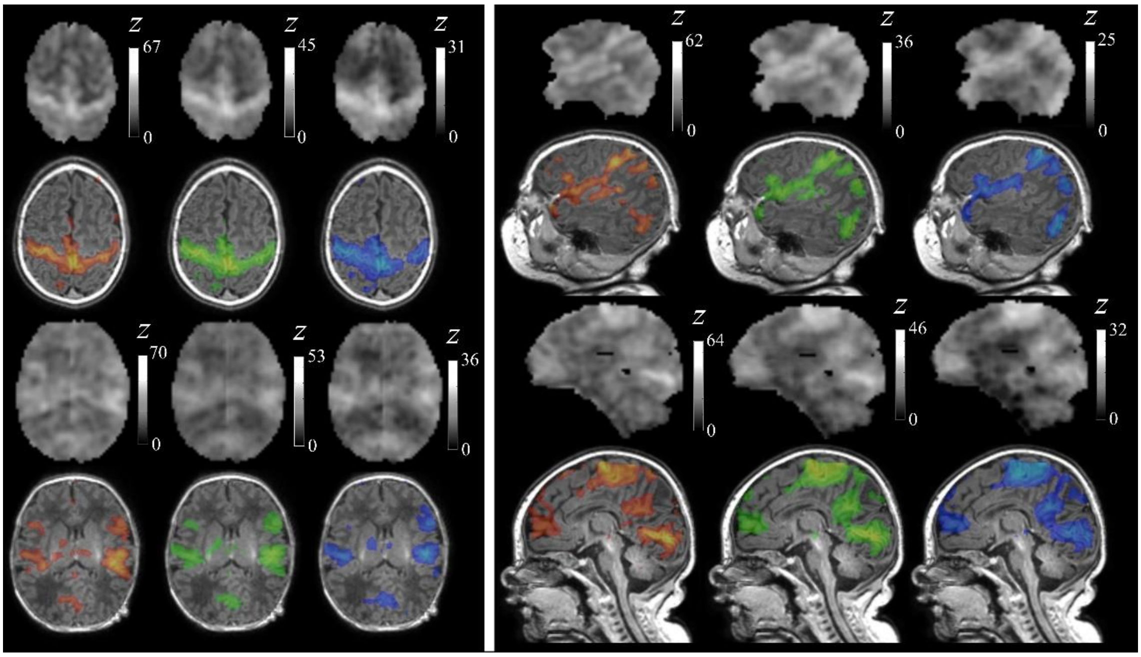

Supplementary Figure 1. Examples of individual results from different cases. The maps correspond to z-transformed Iso-Distant Average Correlation (IDAC) measures for short (1-4mm), middle (4-7mm) and long (7-10mm) distance intervals. The areas with higher functional connectivity (above brain mean z value plus one standard deviation) are emphasized using color display (red for the iso-distance interval 1-4mm, green 4-7mm and blue 7-10mm). The right hemisphere is shown in sagittal images and in the right side of axial images.

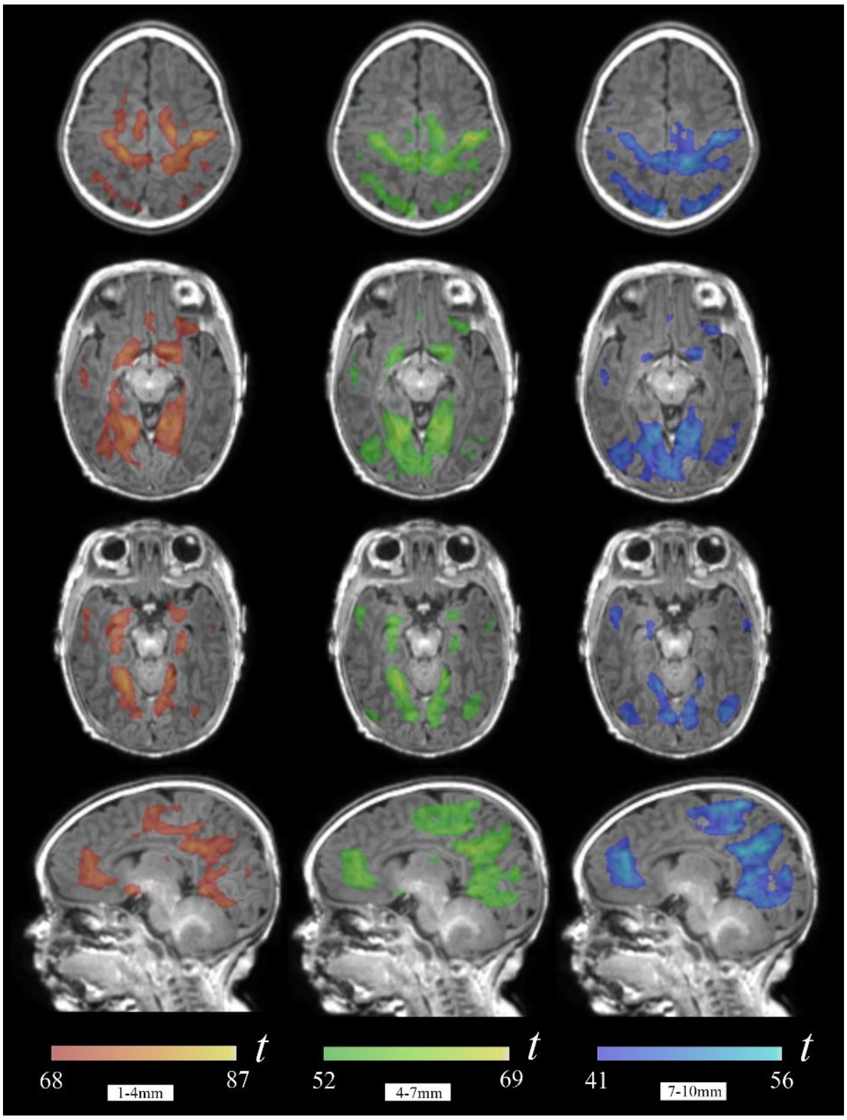

Supplementary Figure 2. Group one-sample t maps for short (1-4mm), middle (4-7mm) and long (7-10mm) local functional connectivity. The figure complements main Figure 2 by showing brain regions with high functional connectivity values at least in 50% of cases in the individual analysis. Brain areas with connectivity values above brain mean plus one standard deviation are shown. The right hemisphere is shown in sagittal images and in the right side of axial images.

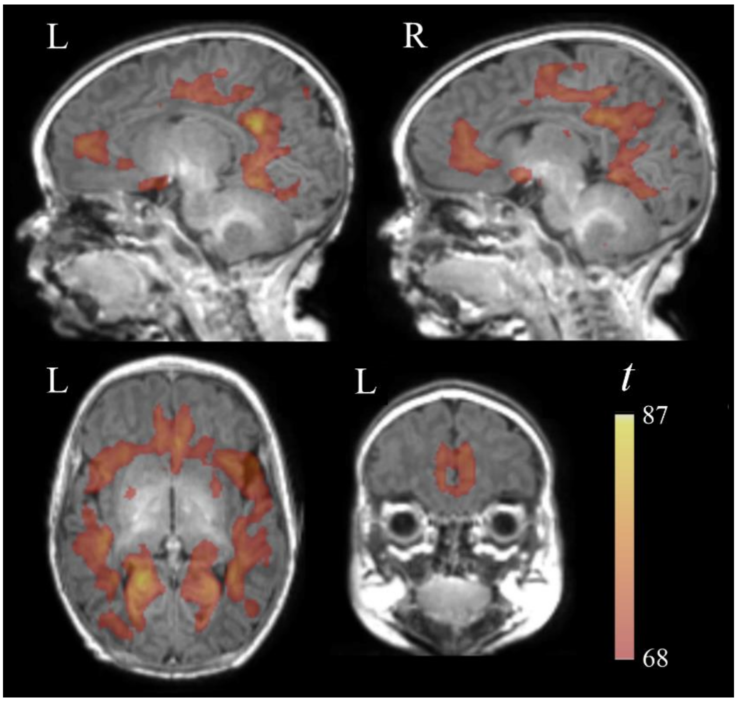

Supplementary Figure 3. Brain slices from the short-distance map (1-4mm) illustrating the differences in functional connectivity between the right (R) and left (L) anterior cingulate cortex.

Supplementary Tables

Supplementary Table 1. Individual analysis in native space

	Connectivity Distance		
	1-4 mm<br>% (n)	4-7 mm<br>% (n)	7-10 mm<br>% (n)
Sensorimotor	100% (61)	100% (61)	100% (61)
Paracentral Lobule	85% (52)	87% (53)	87% (53)
Visual Cortex	100% (61)	100% (61)	100% (61)
MT	100% (61)	100% (61)	97% (59)
Auditory Cortex	100% (61)	98% (60)	95% (58)
Posterior Insula	100% (61)	89% (54)	43% (26)
Frontal Operculum	100% (61)	98% (60)	90% (55)
Anterior Insula	82% (50)	67% (41)	46% (28)
Posterior Cingulate Cortex	98% (60)	97% (59)	85% (52)
Precuneus	100% (61)	100% (61)	87% (53)
Anterior Medial Frontal Cortex	95% (58)	93% (57)	93% (57)
Parietal Association Cortex	70% (43)	72% (44)	72% (44)
R Anterior Cingulate Cortex	72% (44)	46% (28)	20% (12)
L Anterior Cingulate Cortex	21% (13)	16% (10)	8% (5)
Olfactory Cortex	79% (48)	46% (28)	13% (8)
Amygdala	54% (33)	36% (22)	18% (11)
Anterior Hippocampus	57% (35)	33% (20)	18% (11)

Regions with Iso-Distance Average Correlation- IDAC values 1SD above IDAC brain mean

Supplementary Table 2. Significant distance effects in the group analysis.

	**Main Effect of Distance**		**Paired t-test**		
			**Short > Long**		
	x y z	***F***	***x y z***	***t***	***p***
R/L Post. Auditory Cortex/Post. Insula	25 -26 13	106.0	25 -26 13	14.3	< 0.00001
	-21 -28 11	105.5	-21 -28 11	14.5	< 0.00001
R/L Posterior Cingulate Cortex	3 -22 19	28.1	3 -22 19	7.4	< 0.00001
	-3 -24 19	62.7	-3 -24 19	10.8	< 0.00001
R/L Anterior Cingulate Cortex	3 8 -1	125.2	3 8 -3	14.8	< 0.00001
	-1 16 3	70.0	-1 16 1	10.3	< 0.00001
R/L Olfactory Cortex	7 6 -7	18.9	7 6 -7	6.0	< 0.00001
	-7 8 -7	34.5	-7 8 -7	7.6	< 0.00001
R/L Amygdala	13 4 -11	24.0	13 4 -11	6.6	< 0.00001
	-13 2 -11	62.6	-13 2 -11	10.8	< 0.00001
R/L Anterior Hippocampus	19 -10 -15	57.4	19 -10 -15	10.4	< 0.00001
	-13 -10 -19	49.3	-13 -8 -17	9.7	< 0.00001
			**Long > Short**		
Visual Cortex	1 -52 -3	50.0	1 -52 -3	9.2	< 0.00001
Precuneus	3 -40 19	49.1	3 -40 19	9.4	< 0.00001
Anterior Medial Frontal Cortex	3 28 11	42.5	3 28 11	8.8	< 0.00001
Paracentral Lobule	7 -34 43	93.7	7 -34 43	13.0	< 0.00001
R/L Sensorimotor Cortex	25 -26 41	42.0	35 -20 37	6.0	< 0.00001
	-21 -28 41	48.3	-21 -28 41	9.0	< 0.00001
R/L Opercular Region	37 -8 13	33.6	37 -8 13	7.9	< 0.00001
	-35 -14 15	34.7	-35 -14 15	8.0	< 0.00001

x y z coordinates refer to distance (mm) from the anterior commissure. R, right. L, left.

Supplementary Table 3. Significant distance effects in the group analysis, with and without adjusting for age and sex.

	Paired t-test					
	Short > Long					
	x y z	*t*	*p*	*Adj. t* Age[a]	*Adj. t* Sex	*Adj. t* Age & Sex
R/L Post. Auditory Cortex/Post. Insula	25 -26 13	14.3	< 0.00001	14.3	14.5	14.5
	-21 -28 11	14.5	< 0.00001	14.4	14.5	14.5
R/L Posterior Cingulate Cortex	3 -22 19	7.4	< 0.00001	7.5	7.5	7.5
	-3 -24 19	10.8	< 0.00001	10.8	10.9	10.9
R/L Anterior Cingulate Cortex	3 8 -3	14.8	< 0.00001	14.8	14.8	14.8
	-1 16 1	10.3	< 0.00001	10.3	10.3	10.3
R/L Olfactory Cortex	7 6 -7	6.0	< 0.00001	6.0	6.0	6.0
	-7 8 -7	7.6	< 0.00001	7.6	7.7	7.8
R/L Amygdala	13 4 -11	6.6	< 0.00001	6.6	6.6	6.6
	-13 2 -11	10.8	< 0.00001	10.9	11.0	11.1
R/L Anterior Hippocampus	19 -10 -15	10.4	< 0.00001	10.4	10.4	10.4
	-13 -8 -17	9.7	< 0.00001	9.7	9.7	9.6
	Long > Short					
Visual Cortex	1 -52 -3	9.2	< 0.00001	9.3	9.2	9.3
Precuneus	3 -40 19	9.4	< 0.00001	9.4	9.4	9.4
Anterior Medial Frontal Cortex	3 28 11	8.8	< 0.00001	8.8	8.8	8.8
Paracentral Lobule	7 -34 43	13.0	< 0.00001	13.1	13.0	13.1
R/L Sensorimotor Cortex	35 -20 37	6.0	< 0.00001	6.0	6.0	6.0
	-21 -28 41	9.0	< 0.00001	9.0	8.9	8.9
R/L Opercular Region	37 -8 13	7.9	< 0.00001	7.8	7.9	7.9
	-35 -14 15	8.0	< 0.00001	8.1	8.0	8.2

x y z coordinates refer to distance (mm) from the anterior commissure. R, right. L, left. [a]Postnatal age at time of MRI scan. Adjusted models (*Adj.*) were performed as separate analysis in each case.
